# Image Quality Evaluation of Light Field Image Based on Macro-Pixels and Focus Stack

**DOI:** 10.3389/fncom.2021.768021

**Published:** 2022-01-20

**Authors:** Chunli Meng, Ping An, Xinpeng Huang, Chao Yang, Yilei Chen

**Affiliations:** Key Laboratory for Advanced Display and System Application, Shanghai Institute for Advanced Communication and Data Science, School of Communication and Information Engineering, Shanghai University, Shanghai, China

**Keywords:** light field, objective image quality assessment, focus stack, macro-pixels, corner

## Abstract

Due to the complex angular-spatial structure, light field (LF) image processing faces more opportunities and challenges than ordinary image processing. The angular-spatial structure loss of LF images can be reflected from their various representations. The angular and spatial information penetrate each other, so it is necessary to extract appropriate features to analyze the angular-spatial structure loss of distorted LF images. In this paper, a LF image quality evaluation model, namely MPFS, is proposed based on the prediction of global angular-spatial distortion of macro-pixels and the evaluation of local angular-spatial quality of the focus stack. Specifically, the angular distortion of the LF image is first evaluated through the luminance and chrominance of macro-pixels. Then, we use the saliency of spatial texture structure to pool an array of predicted values of angular distortion to obtain the predicted value of global distortion. Secondly, the local angular-spatial quality of the LF image is analyzed through the principal components of the focus stack. The focalizing structure damage caused by the angular-spatial distortion is calculated using the features of corner and texture structures. Finally, the global and local angular-spatial quality evaluation models are combined to realize the evaluation of the overall quality of the LF image. Extensive comparative experiments show that the proposed method has high efficiency and precision.

## Introduction

Light field (LF) imaging technology is designed to record rich scenario information. Compared with ordinary two-dimensional (2D) images and binocular stereoscopic images, LF images are favored in researches like immersive stereoscopic display and object recognition because of their particular characteristics of dense view and post-focusing (Huang et al., 2016; Ren et al., 2017a). For these applications, image quality degradation will directly affect the perception of the immersive experience and the accuracy of object recognition. However, the quality assessment of LF images is different from that of ordinary image types. It involves analyzing the complex imaging structure relationships among dense multi-view LF images. Therefore, it is beneficial to consider the characteristics of LF images, such as the relationship between dense viewpoints, perception of human eyes to the structure of multi-view images, to accurately evaluate the quality. Traditional image quality evaluation models are not suitable for LF because they do not consider the special characteristics of LF images. It is of great significance for the development of LF to build an objective quality evaluation model that effectively utilizes the characteristics of LF images.

The characteristics of LF images are reflected in its various expressions. The dense viewpoints of an LF image, hereinafter referred to as subaperture images (SAIs), represent spatial information of the captured scenes from different visual angles. Adjacent SAIs have strong texture similarity, which enables the compression operation to be better realized. Compression algorithms of LF images can alleviate the problem of inconvenience in transmission caused by a large amount of data of LF images. Furthermore, the reconstruction algorithms play an excellent role in recovering the loss of spatial resolution or angular resolution in the LF image processing. The compression and reconstruction algorithms are mainly based on the multiple representations of LF images: hexagonal lenslet image, rectangular decoded image, SAIs, focus stack, and epipolar plane images (EPIs) (Huang et al., 2019a; Wu et al., 2019). All of the above representations can reflect the angular and spatial characteristics of LF images. Although both compression and reconstruction operations promote the practical application of LF images, they inevitably bring the problem of quality degradation. Moreover, the performance of these algorithms varies a lot, so the criteria to check out the optimal one are necessary.

For situations where SAIs are used to evaluate the quality of LF images, Tian et al. (2018) presented a multi-order derivative feature-based model using the multi-order derivative features extracted on the SAIs of LF images. However, their analysis remains in the texture aspect of spatial information, lacking the analysis of the connection between the angular and spatial information. As an LF image can be regarded as a low-rank 4D tensor, Shi et al. (2019) adopted the tensor structure of the cyclopean image array from the LF to explore the angular-spatial characteristic. Zhou et al. (2020) used tensor decomposition of view stack in four directions to extract the spatial-angular features. To explore the angular-spatial characteristics of LF images, Min et al. (2020) averaged the structural matching degree of all viewpoints to compute the spatial quality and analyzed the amplitude spectrum of near-edge mean square error along viewpoints to express the angular quality. Xiang et al. (2020) computed the mean difference image from SAIs to describe the depth and structural information of LF images, and it used a curvelet transform to reflect the multi-channel characteristics of the human visual system.

The focus stack is constructed by stacking the refocused images from the perspective of depth, which reflects both the texture and depth information of LF images. Meng et al. (2019) compared different objective metrics under SAIs and the focus stack, which verified the superiority of the refocus characteristic of LF images. Meng et al. (2019) utilized the LF angular-spatial and human visual characteristics and verified the effectiveness of the assumed optimal parallax range. Meng et al. (2021) built a key refocused image extraction framework based on the maximal spatial information contrast and the minimal angular information variation to reduce the redundancy of quality evaluation in the focus stack.

The depth feature makes the LF more popular in object detection, three-dimensional reconstruction, and other applications. Paudyal et al. (2019) compared different depth extraction strategies and assessed the quality of LF through the structural similarity of the depth map. It is proven that the depth information is effective in reflecting the distortion degree of LF images, but Paudyal et al. (2019) ignored the texture structure information of LF images. Therefore, some studies have attempted to combine depth features with the features from SAIs to achieve better prediction results. Shan et al. (2019) combined the ordinary 2D features of SAIs and sparse gradient dictionary of LF depth map. Tian et al. (2020) performed radial symmetric transformation on the luminance components of all dense viewpoints to extract symmetric features and used depth maps to measure the structural consistency between viewpoints, which explored the way humans perceive structures and geometries.

To preferably explore the angular-spatial characteristics of LF, many pieces of research are devoted to take advantage of various LF expressions. For the form of uniting multiple representations, Luo et al. (2019) used the global entropy and uniform local binary pattern features of a lenslet image to evaluate the angular consistency, and adopted the information entropy of SAIs to measure spatial quality. Fang et al. (2018) calculated the change in visual quality by combining the gradient amplitude of SAIs and EPIs.

In addition to traditional methods, as deep learning exhibits excellent performance in other aspects of image processing, some teams have worked to fill the research gap of deep learning in the quality evaluation of LF images. Zhao et al. (2021) proposed an LF-IQA method based on the multi-task convolutional neural network (CNN), in which the EPI patches were taken as the input of the CNN model and the model followed ResNet in the convolution layer. Lamichhane et al. (2021) proposed an LF-IQA metric based on a CNN that measures the distortion of the saliency map. Lamichhane et al. (2021) confirmed that there is a strong correlation between the distortion levels of normalized images and the corresponding saliency maps. Guo et al. (2021) proposed a deep neural network-based approach, in which the relationship among SAIs was obtained by SAI fusion and global context perception models. To solve the problem of insufficient databases, they proposed a ranking-based method to generate pseudo-labels to pre-train the quality assessment network, and then fine-tuned the model at small-scale data sets with real labels.

**Figure 1:**
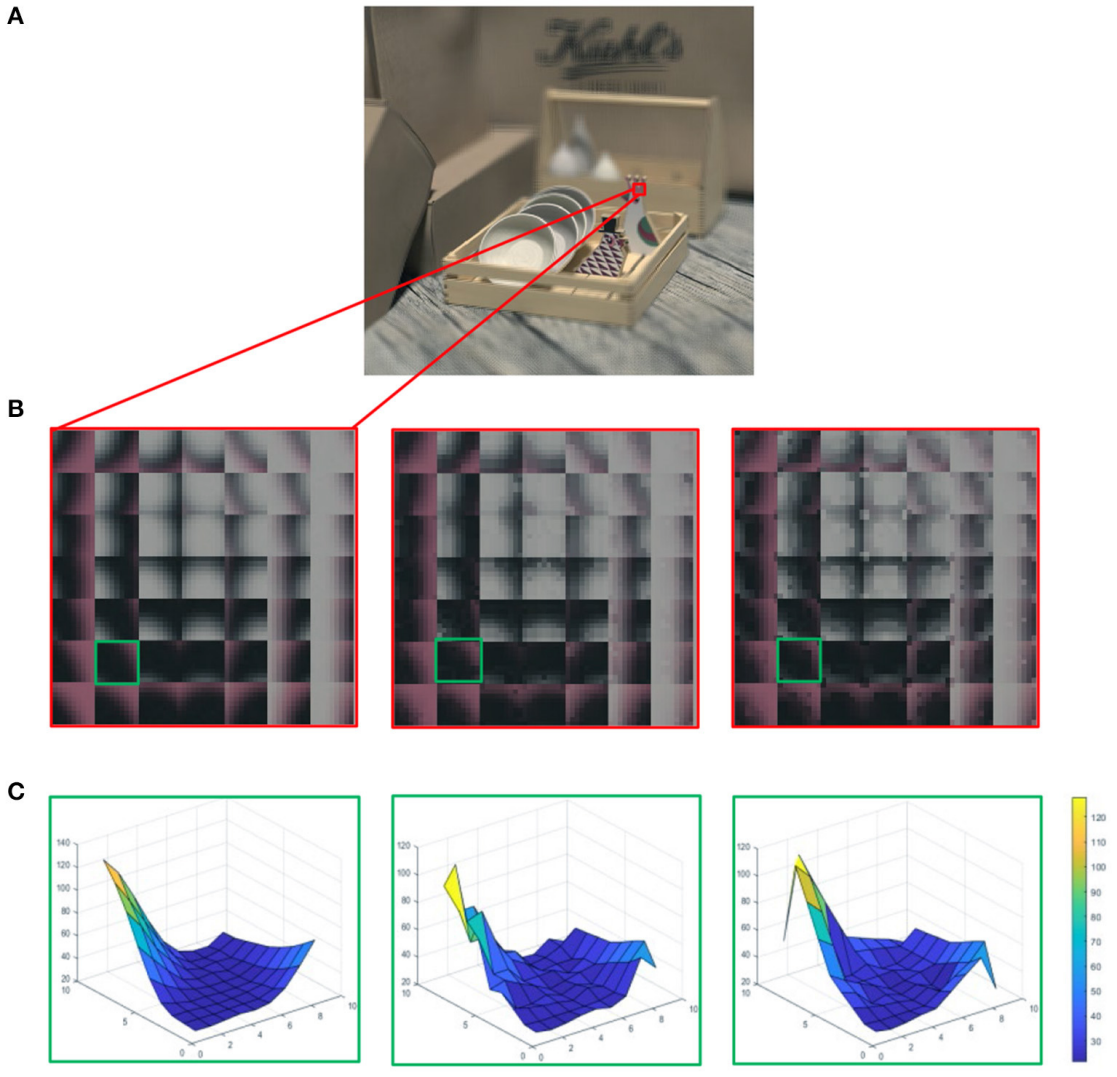
**(A)** The referenced light field (LF) image in the form of decoded lenslet. **(B)** The first column is the enlarged local macro-pixels from **(A)**, and the other two columns correspond to macro-pixels with different degrees of distortion, which increased from left to right. **(C)** Each column corresponds to the grid distribution of gray values of a single macro-pixel in the green block in **(B)**.

This paper attempts to build a quality evaluation index that comprehensively considers the angular-spatial characteristics of LF images and human vision characteristics. The angular information of LF is directly expressed in the form of macro-pixel, which has been widely used in LF compression (Schiopu and Munteanu, 2018). Macro-pixels can be simply used to compare changes in angular information and do not involve a complex analysis of texture. For lenslet images, the array of pixels beneath each microlens is named as a macro-pixel. As shown in Figure 1, the second line is the enlarged local macro-pixels of the referenced lenslet image and the corresponding distorted macro-pixels. The enlarged part of the lenslet image contains 7 × 7 macro-pixels, and each macro-pixel contains 9 × 9 pixels. It can be seen from Figure 1C that luminance and chrominance have changed in the distorted macro-pixels. Hence, we first utilize the angular information of all spatial positions to globally analyze the angular-spatial quality of LF images. As for spatial information, texture structure is an important and a direct means for human eyes to perceive image quality. Ingeniously, the focus stack not only reflects the texture structure information but also partly maps the angular information. Min et al. (2018) mentioned that quality degradations can cause local image structure changes, and Min et al. (2017a,b) mentioned that corners and edges are presumably the most important image features that are sensitive to various image distortions. Therefore, we construct a local LF angular-spatial quality evaluation model based on the focus stack through the measurement of corner and texture structures. Finally, the abovementioned two clues are combined to represent the overall quality of LF images. The contributions of this paper mainly include the following three points.

• A prediction framework of global angular-spatial distortion of LF images is established on the lenslet images. First, the distortion of angular information is calculated by averaging the changes in luminance and chrominance of each macro-pixel. All the evaluated values are arranged according to the corresponding spatial coordinates, forming an array of predicted values of angular distortion. Then, the visual saliency of the central SAI, which reflects the spatial information distribution with human visual characteristics, is introduced to pool an array of predicted values of angular distortion to obtain the predicted value of global distortion.

• An evaluation framework of local angular-spatial distortion of LF images is built on the principal components of the focus stack. The loss of the focalizing structure and the distortion of spatial texture structure are analyzed on the principal components through the corner similarity and texture similarity, respectively. The final local distortion is evaluated by fusing the predicted values of the focalizing structure and texture structure.

• The proposed method is compared with multiple objective metrics in the stitched multi-view image framework, and their results are analyzed with three subjective LF-IQA databases to verify their effectiveness and robustness.

## Materials and Methods

Although the angular-spatial characteristics of LF are reflected in various expressions of LF, it is still a great challenge to extract and calculate the angular-spatial characteristics of LF. The lenslet images not only macroscopically reflect the global angular-spatial information of the LF images, but also microscopically reflect the angular information distribution. Inspired by this, we intend to start from the macro-pixels of the lenslet images to evaluate the angular distortion at the micro level, and then use the feature of spatial information to pool the predicted values of angular distortion. In consideration of the lack of analysis of useful texture and edge structure in the scene, which has a great influence on the quality perception, in the calculation of global distortion of LF images, the study in this paper will combine with other LF representations to supplement its deficiency. As each refocused image in the focus stack contains both angular-spatial information and texture structure, this paper chooses to analyze the texture and edge structure of the LF images with the focus stack.

**Figure 2:**
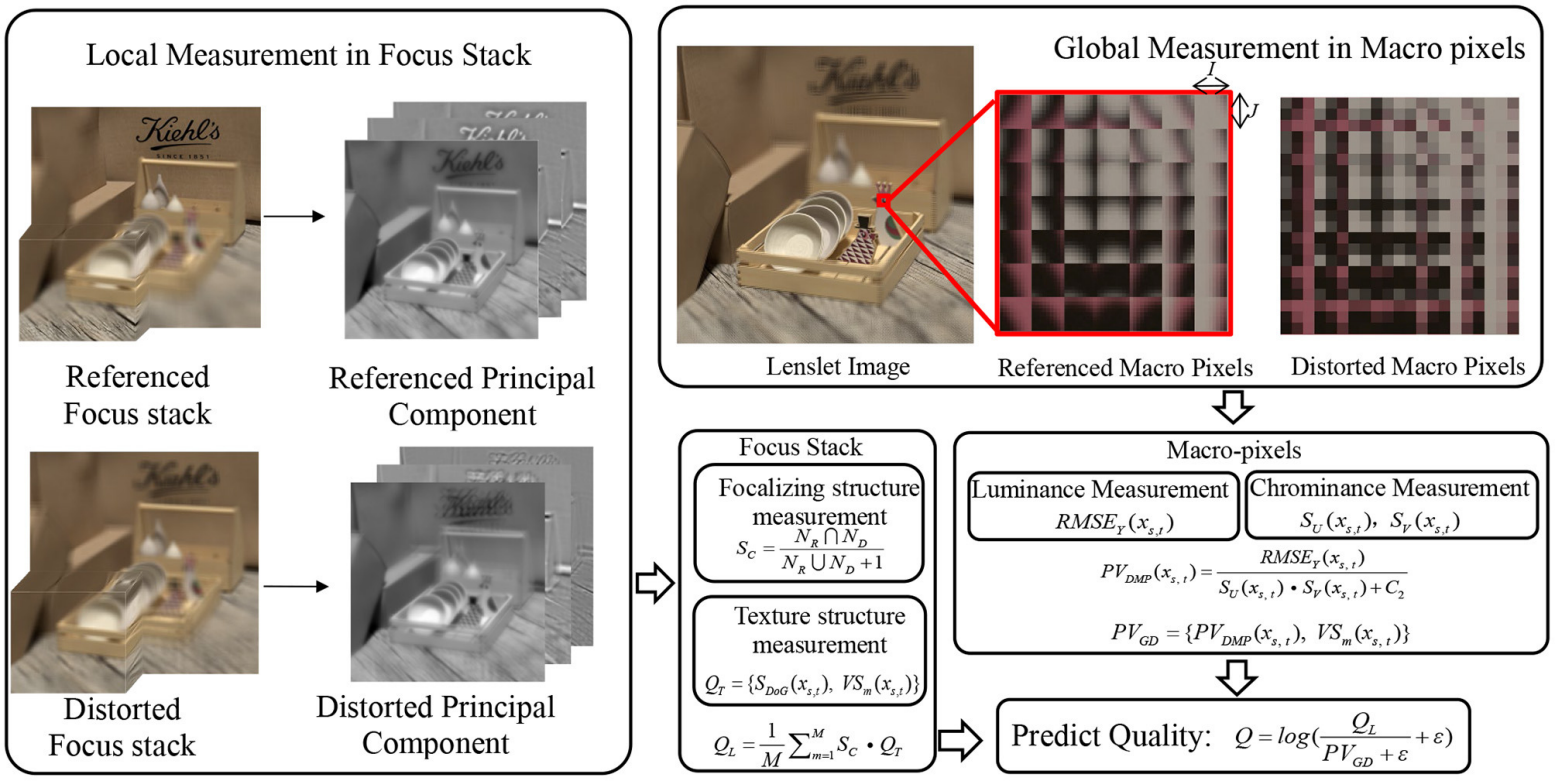
The proposed LF-IQA framework based on the angular-spatial feature information.

According to the abovementioned analysis, we propose an evaluation method to comprehensively predict the distortion of LF images from both global and local aspects. The distribution of global and local distortion is analyzed from the lenslet images and focus stack, respectively. As illustrated in Figure 2, the global distortion in lenslet images is analyzed at each macro-pixel through the luminance and chroma channels. After then, we utilize the visual salient feature of spatial information to assign different weights to the measured values of each distorted macro-pixel, so as to realize the fusion of spatial information and angular information. Moreover, human visual characteristic has been taken into account in the calculation of visual saliency. As the single macro-pixel of a lenslet image lacks the texture and edge information of the objects in the scene, we complement the global distortion measurement by analyzing the principal components in the focus stack. The prediction processes of global and local distortion are described in sections The Prediction of Global Angular-Spatial Distortion and The Evaluation of Local Angular-Spatial Quality, respectively, and the two complementary prediction frameworks are fused in section The Evaluation of Union Angular-Spatial Quality.

### The Prediction of Global Angular-Spatial Distortion

A lenslet image is composed of an array of macro-pixels embedded with angular information. The array of macro-pixels reflects the distribution of angular-spatial distortion macroscopically, while a single macro-pixel reflects the distribution of angular distortion microscopically. The size of a lenslet image is *S* × *T* units of macro-pixels, and the size of a macro-pixel is *I* × *J*, where *S* × *T* is the spatial resolution of LF images, and *I* × *J* is the angular resolution of LF images.

As it can be seen from Figures 1A,B, the distortion of macro-pixels is manifested as the changes in luminance and chrominance. Figure 1C describes the grid distribution of referenced and different distorted macro-pixels, which reflects the influence of distortion on the angular information. Considering that a single macro-pixel involves all the angular information of the corresponding spatial position, we first compute the angular distortion within each macro-pixel.

As a single macro-pixel does not involve the complex texture and edge structure of the objects in the scene, we decided to study the variation of luminance information and chroma information in each macro-pixel. Without considering the image texture structure information, the root mean squared error (*RMSE*) method can simply and accurately calculate the error between referenced and distorted macro-pixels. As people are more sensitive to the change of luminance than that of chrominance (Su, 2013), we mainly measure the distortion of each macro-pixel on the luminance channel. Specifically, Equation (1) expresses the *RMSE* of luminance (*RMSE*_*Y*_) of the referenced macro-pixel (*Y*_*R*_) and the distorted macro-pixel (*Y*_*D*_):


(1) $$\begin{array}{l} RMSE_{Y}\left(x_{s,t}\right)=\sqrt{\frac{1}{I▪J}\left(\sum_{i=\text{1}}^{I}\sum_{j=\text{1}}^{J}{\left(Y_{R}\left(x_{i,j}\right)-Y_{D}\left(x_{i,j}\right)\right)}^{\text{2}}\right)} \end{array}$$

where *x*_*s,t*_ is the pixel value on the spatial coordinate (*s, t*). *x*_*i,j*_ is the pixel value on the angular coordinate (*i, j*). *I* and *J* are the angular resolutions, in this paper, *I* = 9, *J* = 9.

In addition to the variation of luminance information in the macro-pixel array, the distortion of chroma information will also affect the perception of the overall quality of images. As macro-pixels have no texture and edge structure of objects in the scene, the measurement of chroma distortion of macro-pixels can be simpler and more direct. Considering that the chrominance information has a much smaller impact on the overall quality than the luminance, we adopt the similarity measurement method that is widely used in objective assessment methods, as given in Equations (2) and (3). The chrominance information is analyzed in the YUV color space. The similarity map of each macro-pixel is averaged to calculate the quality value of the corresponding spatial position (*s, t*).


(2) $$\begin{array}{l} S_{U}\left(x_{s,t}\right)=\frac{1}{I▪J}\left(\sum_{i=\text{1}}^{I}\sum_{j=\text{1}}^{J}\frac{2U_{R}\left(x_{i,j}\right)\;▪\;U_{D}\left(x_{i,j}\right)+C_{1}}{{U_{R}}^{2}\left(x_{i,j}\right)+{U_{D}}^{2}\left(x_{i,j}\right)+C_{1}}\right) \end{array}$$

(3) $$\begin{array}{l} S_{V}\left(x_{s,t}\right)=\frac{1}{I▪J}\left(\sum_{i=\text{1}}^{I}\sum_{j=\text{1}}^{J}\frac{2V_{R}\left(x_{i,j}\right)\;▪\;V_{D}\left(x_{i,j}\right)+C_{1}}{{V_{R}}^{2}\left(x_{i,j}\right)+{V_{D}}^{2}\left(x_{i,j}\right)+C_{1}}\right) \end{array}$$

where *S*_*U*_ and *S*_*V*_ are the color similarity of *U* and *V* channels. *U*_*R*_ and *V*_*R*_ are referenced macro-pixels of *U* and *V* channels, and *U*_*D*_ and *V*_*D*_ are distorted macro-pixels of *U* and *V* channels. The constant *C*_1_ is used to maintain the stability of the similarity measurement function (Zhang et al., 2011), we fixed *C*_1_ = 1 through the experiments.

The smaller *RMSE*_*Y*_ between the referenced and distorted macro-pixel signifies the smaller error of the luminance components between them, while the greater chrominance similarity represents the smaller chroma error. For each macro-pixel, we use Equation (4) to fuse the predicted values of luminance and chrominance components. The values of *RMSE*_*Y*_ are in the range of 0–255, to make the contribution of chroma less to the overall distortion prediction than the luminance, we set *C*_2_ to 0.01, so that the range of chroma error is 0.99–100.


(4) $$\begin{array}{l} PV_{DMP}\left(x_{s,\;t}\right)=\frac{RMSE_{Y}\left(x_{s,\;t}\right)}{S_{U}\left(x_{s,\;t}\right)\;▪\;S_{V}\left(x_{s,\;t}\right)+C_{2}} \end{array}$$

where *PV*_*DMP*_(*x*__*s*_, *t*_) is the fused prediction value of the distorted macro-pixel in the spatial coordinate (*s, t*), sϵ[1, *S*], tϵ[1, *T*]. *S* and *T* are the spatial resolution, in this paper, *S* = 434, *J* = 625. The *PV*_*DMP*_ values arranged in spatial coordinates form an array of predicted values of angular distortion.

To integrate the angular information and spatial information of LF images in the process of image quality assessment, we intend to pool the predicted values of angular distortion using the spatial information. The exciting thing is that the corresponding spatial coordinates of macro-pixels reflect the significance of the texture and contour of the LF images. As the central SAI is the main perspective from which humans observe the scenes, we choose to use the features of the central SAI to pool an array of predicted values of angular distortion. The visual saliency map of the central SAI, which reflects the spatial information distribution with human visual characteristics, is introduced to pool the predicted values of all distorted macro-pixels, as given in Equation (5):


(5) $$\begin{array}{l} PV_{GD}=\frac{\sum_{s\text{=}1}^{S}\sum_{t\text{=}1}^{T}PV_{DMP}\left(x_{s,\;t}\right)\;▪\;VS_{m}\left(x_{s,\;t}\right)}{\sum_{s\text{=}1}^{S}\sum_{t\text{=}1}^{T}VS_{m}\left(x_{s,\;t}\right)} \end{array}$$

where *PV*_*GD*_ is the predicted value of global angular-spatial distortion of LF images. VS_*m*_ (*x*_*s,t*_) = max [*VS*_*r*_(*x*_*s,t*_), *VS*_*d*_(*x*_*s,t*_)], *VS*_*r*_(*x*_*s,t*_), and *VS*_*d*_(*x*_*s,t*_) are visual saliency maps of the central SAIs of referenced and distorted LF images, respectively. In this paper, we use the simple saliency model in Zhang et al. (2013), which integrates the frequency prior, color prior, and location prior and has been proven to be a simple and an effective visual saliency model that simulates the perceptual characteristics of human eyes to the images (Zhang et al., 2014).

### The Evaluation of Local Angular-Spatial Quality

As mentioned earlier, the prediction of global angular-spatial distortion lacks direct measurements of the texture and edge structure of the objects in the scenes. This section aims to complement the global distortion measurement by analyzing the principal components in the focus stack. The focus stack consists of a series of refocused images arranged in the direction of depth. A refocused image is obtained by shifting and summing the SAIs at a given slope. Therefore, the refocused images only contain the local angular-spatial information of LF images. Specifically, the distortion of the angular information is directly manifested as the loss of the focalizing structure in the focus stack, while the distortion of the spatial information is manifested as various forms of destruction of the texture and edge structure in the scenes.

**Figure 3:**
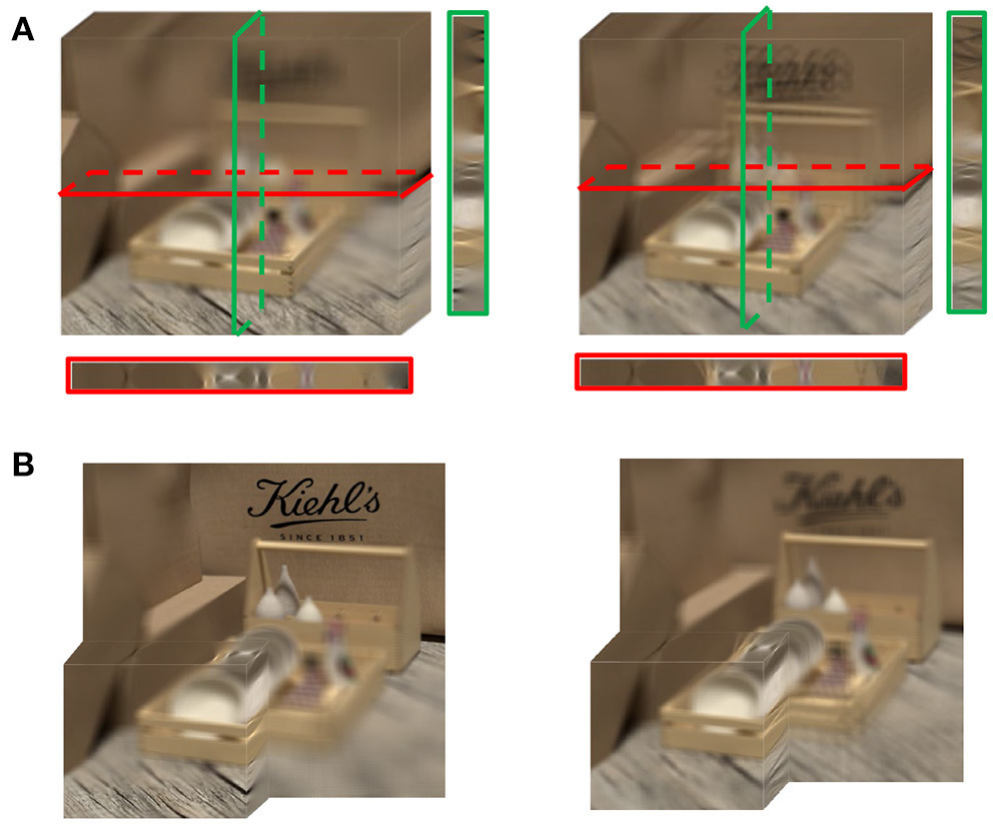
Focus stack. **(A)** The focus stack of reference and distortion from left to right. The red and green boxes are the cross and vertical sections of the focus stack, respectively. **(B)** The partial focus stack of reference and distortion from left to right.

The loss of the focalizing structure is reflected as the disorder of the focus state. As shown in Figure 3A, the red and green boxes correspond to the cross and vertical sections of the focus stack. The sections of the referenced focus stack show that the focalizing structure is orderly, while the focusing state of the distorted focus stack is chaotic. Specifically, the foremost focusing position of the referenced focus stack is located on the wood plate, while the forefront refocused slice of the distorted focus stack is not in the focus state. Moreover, Figure 3B shows that the backmost refocus slice of the referenced focus stack focused on the text, while the corresponding distorted refocus slice was not focused on the text that should be focused due to the angular-spatial distortion. In a word, the energy distribution of the distorted focus stack is scattered throughout the whole depth range, and the original focalizing structure is destroyed.

**Figure 4:**
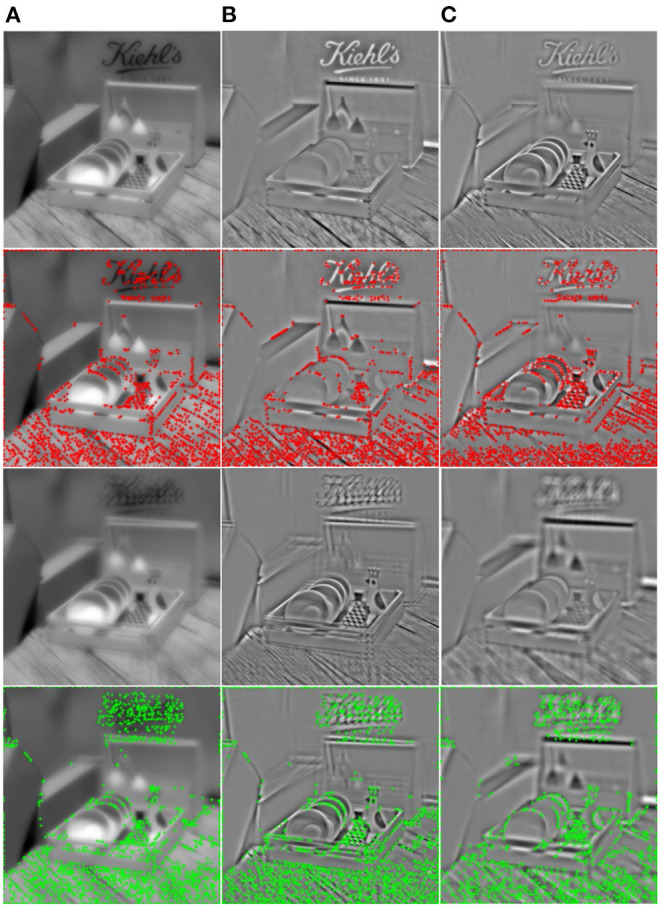
The first and third rows are the principal components of the referenced and distorted focus stack, respectively. The second and fourth rows are the corners of referenced and distorted principal components, respectively. **(A)** The first principal component; **(B)** the second principal component; and **(C)** the third principal component.

We also noticed from Figure 3 that there is a defocused blur in the unfocused parts of the focus stack. When human eyes focus on a point of the scene, the object points at other depths of the field become blurred. The focus stack simulates the human eyes' habit of viewing a scene, so a defocused blur is inevitably introduced. To alleviate the effect of a defocused blur, we attempt to use principal component analysis (PCA) to extract the main components from the focus stack, as shown in the first and third rows of Figure 4. As we have analyzed the effect of chrominance on the prediction of global distortion (section Databases for Validation), the principal components are extracted only in the grayscale of the focus stack (Ren et al., 2017b).

Principal component analysis is a means of dimension reduction. The advantage is that PCA not only reduce the calculation amount for the focus stack but also alleviate the influence of a defocused blur in the analysis of the focalizing structure. By sorting the eigenvalues and corresponding eigenvectors of the covariance matrix of gray refocused slices in the focus stack, the focus stack can be rearranged according to the proportion of information content. As for the number of selected principal components, the experimental comparison and analysis are conducted (section 4.6). In this paper, the first three principal components are selected to predict the local angular-spatial quality for accuracy and simplicity.

For the principal components of the focus stack, we analyze the loss of focalizing structure and texture damage caused by the angular-spatial distortion. Firstly, the corner structure based on phase congruency (PC-corner) is used to evaluate the focalizing structure loss. As shown in the second and fourth rows of Figure 4, the PC-corner operator detects the features as points in an image with a high-phase component order in the Fourier domain, and it is not affected by luminance, contrast, and scale. The PC-corner feature operator can detect a wide range of features, such as angle, line, and texture information of images.

The corner response function is developed based on the covariance matrix of PC (Kovesi, 2003), as given in Equation (6):


(6) $$\begin{array}{l} CM=\left[\begin{array}{cc} P{C_{x}}^{2} & PC_{x}▪PC_{y}\\ PC_{x}▪PC_{y} & P{C_{y}}^{2} \end{array}\right] \end{array}$$

where *PC*_*x*_ and *PC*_*y*_ are PC-corner at horizontal and vertical directions. The phase consistency utilizes the log-Gabor filter of multi-scale and multi-direction. The final covariance matrix is normalized with the orientations used in the log-Gabor filter. In this paper, we use three scales (*n* = 1, 2, 3) and six orientations (θ = 0, π/6, π/3, π/2, 2π/3, 5π/6).

Being different from the structural loss of ordinary image, the structural loss of the focus stack includes the reduction and increment of structure due to the angular-spatial distortion. Therefore, we use the form of Equation (7) to calculate the corner similarity *S*_*C*_ between referenced and distorted principal components.


(7) $$\begin{array}{l} S_{C}=\frac{N_{R}⋂N_{D}}{N_{R}⋃N_{D}+1} \end{array}$$

where *N*_*R*_ and *N*_*D*_ are the number of corners in referenced and distorted principal components, respectively. ∩ is the intersection of *N*_*R*_ and *N*_*D*_, and ∪ is the union of *N*_*R*_ and *N*_*D*_. The constant 1 is added to avoid the denominators being 0.

Secondly, in addition to assessing the loss of the focalizing structure, the angular-spatial distortion can also lead to an obvious texture damage of the focus stack. Similar to the evaluation of focalizing structure, the prediction of texture distortion is conducted on the principal components of the focus stack. The vertebrate retina can be mathematically represented by the Laplacian of Gaussian, which is an effective method of texture calculation reflecting the characteristics of human vision. Considering that the waveform distribution of DoG algorithm is similar to that of Laplacian of Gaussian, and the complexity of DoG is much smaller, we choose DoG to calculate the texture feature.

The DoG is the difference of the image signal *I*(*x*__*s*_, *t*_) convolved with the two different Gaussian scales σ1, σ2:


(8) $$\begin{array}{l} L\left(x_{s,t},\sigma_{1}\right)=G\left(x_{s,t},\sigma_{1}\right)*I\left(x_{s,t}\right) \end{array}$$

(9) $$\begin{array}{l} L\left(x_{s,t},\sigma_{2}\right)=G\left(x_{s,t},\sigma_{2}\right)*I\left(x_{s,t}\right) \end{array}$$

(10) $$\begin{array}{l} DoG\left(x_{s,t}\right)=L\left(x_{s,t},\sigma_{1}\right)-L\left(x_{s,t},\sigma_{2}\right) \end{array}$$

where *L* (*x*__*s*_, *t*_, σ_1_) and *L* (*x*__*s*_, *t*_, σ_2_) are convolutions of the image signal *I* (*x*__*s*_, *t*_) with Gaussian functions at the two different Gaussian scales (σ1, σ2).

Equation (11) was initially used in the calculation of structure similarity (SSIM) (Wang et al., 2004), and then widely used for the distance calculation of feature similarity (FSIM) in objective assessment methods. Hence, the texture similarity of referenced and distorted principal components is calculated by Equation (11).


(11) $$\begin{array}{l} S_{DoG}\left(x_{s,t}\right)=\frac{2DoG_{R}\left(x_{s,t}\right)\cdot DoG_{D}\left(x_{s,t}\right)+C_{\text{3}}}{Do{G_{R}}^{2}\left(x_{s,t}\right)+Do{G_{D}}^{2}\left(x_{s,t}\right)+C_{\text{3}}} \end{array}$$

where *DoG*_*R*_ and *DoG*_*D*_ are differences of Gaussian feature of referenced and distorted principal components, respectively. The constant *C*_3_ is used to maintain the stability of the similarity measurement function, we fixed *C*_3_ = 0.1 through the experiments.

Concretely, the similarity map of DoG is pooled through the feature of visual saliency to obtain the quality of texture *Q*_*T*_, as given in Equation (12). The calculation method of visual saliency is the same (as mentioned in section Databases for Validation):


(12) $$\begin{array}{l} Q_{T}=\frac{\sum_{s=1}^{S}\sum_{t=1}^{T}S_{DoG}\left(x_{s,t}\right)▪VS_{m}\left(x_{s,t}\right)}{\sum_{s=1}^{S}\sum_{t=1}^{T}VS_{m}\left(x_{s,t}\right)} \end{array}$$

**Figure 5:**
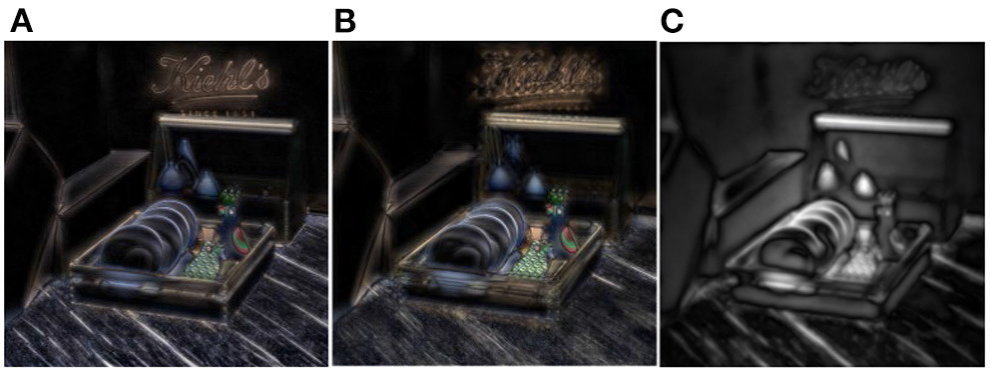
The light flow in the focus stack and the visual saliency map. **(A)** The light flow of referenced focus stack. **(B)** The light flow of distorted focus stack. **(C)** The visual saliency map based on the sum of light flow of focus stack.

We define the light flow in the focus stack as the sum of the differences between adjacent refocus slices. The feature of visual saliency *VS*_*m*_ is computed with the light flow of the focus stack, as shown in Figure 5 and Equation (13).


(13) $$\begin{array}{l} VS_{m}=\max\left(VS_{Lif\text{-}R},VS_{Lif\text{-}D}\right) \end{array}$$

where *VS*_*Lif*−*R*_ and *VS*_*Lif*−*D*_ are visual saliency maps of the light flow of referenced and distorted focus stack, respectively.

Finally, the local angular-spatial quality *Q*_*L*_ is obtained by averaging the fused quality of the focalizing structure and texture. *M* in Equation (14) is the number of principal components, which is analyzed in section 4.6 at different *M* values.


(14) $$\begin{array}{l} Q_{L}=\frac{1}{M}\sum_{m=1}^{M}S_{C}\;▪\;Q_{T} \end{array}$$

### The Evaluation of Union Angular-Spatial Quality

According to sections Databases for Validation and Performance Analysis of Image Quality Metrics, a smaller *PV*_*GD*_ value indicates the smaller global distortion, which corresponds to the higher global quality, while a smaller *Q*_*L*_ value indicates the smaller local quality. The overall quality of LF images is calculated by fusing the predicted value of global angular-spatial distortion *PV*_*GD*_and local angular-spatial quality *Q*_*L*_. Considering that *PV*_*GD*_ and *Q*_*L*_ are inversely and directly proportional to the overall quality, respectively, we use Equation (15) to calculate the overall quality of the LF images.


(15) $$\begin{array}{l} Q=log\left(\frac{Q_{L}}{PV_{GD}+\varepsilon }+\varepsilon \right) \end{array}$$

where log operation is added to increase the linearity of the results, which conforms to the human eyes' ability to recognize the light intensity (Min et al., 2020). *PV*_*GD*_ is given by Equation (5), and *Q*_*L*_ is given by Equation (14). ε is a constant for equation stability, which is set as 0.0001.

## Results

### Databases for Validation

Resource identification initiative. To verify the performance of the proposed method, experiments were conducted on three subjective quality assessment databases of LF images, including the database of traditional distortion types: SHU (Shan et al., 2019), video compression, and LF compression types: VALID-10bit (Viola and Ebrahimi, 2018), and LF reconstruction types: NBU-LF1.0 (Huang et al., 2019b). The detailed information of these databases is listed in Table 1.

1. *SHU database: traditional distortion types*. The SHU database is composed of 8 referenced LF images and 240 distorted LF images. There are five distortion types, including the classical compression artifacts (JPEG and JPEG2000) and other distortions (motion blur, Gaussian blur, and white noise). Each type of distortion has six distortion levels. The database is visualized by pseudo-sequence video of SAIs to the subjects.
2. *VALID-10bit database: video compression and LF compression distortion types*. There are two general compression schemes (HEVC and VP9) and three compression schemes specifically designed for LF (Ahmad et al., 2017; Tabus et al., 2017; Zhao and Chen, 2017). For each compression type, 4 levels of compression are introduced, and a total of 100 compressed LFs are included in this data set. It has five referenced LF contents and is evaluated in the passive methodology. For the passive evaluation, the perspective views were shown as animation and followed by the refocused views (Viola et al., 2017).
3. *NBU-LF1.0 data set: reconstruction distortion types*. It includes five LF reconstruction schemes: neighbor interpolation (NN), bicubic interpolation (BI), learning-based reconstruction (EPICNN), disparity-map-based reconstruction (DR), and spatial super-resolution reconstruction (SSRR). It has 14 referenced LF contents and 210 distorted LF images. Each reconstruction type has three levels of reconstruction.

**Table 1:** The detailed information of LF-IQA databases used in the experiment.

**Database**	**Distortion types**		**Distortion levels**
SHU	Traditional Distortion	JPEG JPEG2000: Motion Blur: Gaussian Blur: White Noise:	QLs: 1, 10, 15, 20, 50, 90 CRs: 10, 70, 150, 200, 250, 400, 600 MLs: 10, 20, 60, 100, 150, 200 SDs: 0.5, 2, 4, 5, 10, 20 SDs: 0.05, 0.1, 0.3, 0.5, 1, 2
VALID-10bit	Video & LF Compression	HEVC, VP9, Ahmad et al., 2017; Tabus et al., 2017; Zhao and Chen, 2017	Bpp: 0.005, 0.02, 0.1, 0.75.
NBU-LF1.0	LF Reconstruction	NN, BI, EPICNN DR VDSR	RFs: 5, 3, 2 RFs: 7, 5, 3 RFs: 2, 3, 4

To reduce the complexity, the number of multiple views selected from the databases in Table 1 is 9 × 9, and the image resolution is 434 × 625.

### Performance Analysis of Image Quality Metrics

There are three main representations of LF with whole global information: EPIs, lenslet images, and SAIs. First of all, the oblique texture structure in EPIs is not similar to the texture structure of objects in ordinary images, which is not conducive to the realization of traditional image quality evaluation methods. Except for the statistical IQA method at pixel-level, such as peak signal-to-noise ratio (PSNR), most traditional image quality evaluation methods cannot take the advantage of their simulation in image structure and human visual characteristics. Secondly, lenslet images have discontinuities of scene texture due to the angular information, which is not conducive to the application of algorithms based on human visual characteristics. Thirdly, SAIs can be regarded as a matrix of 2D images distributed in different angular directions. The superiority of traditional algorithms can be developed in the stitched SAIs, which is due to the fact that the stitched SAIs can be seen as a large 2D image with texture redundancy. Hence, we decide to apply the traditional algorithms to the stitched SAIs to carry out the following comparison experiments.

In general, the objective evaluation includes three categories according to their dependence on the reference image: full reference (FR), reduced reference (RR), and no reference (NR) (Wang and Bovik, 2006). In Table 2, the performance of the proposed MPFS is broadly compared with the classical FR, RR, and NR metrics over three subjective LF-IQA databases. The metrics mainly include classical traditional IQA metrics and the state-of-the-art LF-IQA metrics. 2D FR IQA metrics include PSNR, SSIM (Wang et al., 2004), multi-scale SSIM (MS-SSIM) (Wang et al., 2003), information weighting SSIM (IW-SSIM) (Wang and Li, 2010), FSIM (Zhang et al., 2011), FSIM based on Riesz transforms (RFSIM) (Zhang et al., 2010), noise quality measure (NQM) (Damera-Venkata et al., 2000), gradient similarity (GSM) (Liu et al., 2011), visual signal noise ratio (VSNR) (Chandler and Hemami, 2007), most apparent distortion (MAD) (Larson and Chandler, 2010), gradient magnitude similarity deviation (GMSD) (Xue et al., 2013), and HDRVDP (Mantiuk et al., 2011). Sparse feature fidelity (SFF) (Chang et al., 2013), universal image quality index (UQI) (Wang and Bovik, 2002), visual saliency-induced index (VSI) (Zhang et al., 2014), 2D RR IQA metrics include wavelet-domain natural image statistic model (WNISM) (Wang and Simoncelli, 2005), wavelet-based contourlet transform (WBCT) (Gao et al., 2008), and contourlet (Tao et al., 2009). Multi-view FR IQA metrics include morphological pyramids PSNR (MP-PSNR) (Sandić-Stanković et al., 2015), morphological wavelets PSNR (MW-PSNR) (Sandić-Stanković et al., 2015), MW-PSNRreduc (Sandić-Stanković et al., 2015), and 3DSwIM (Battisti et al., 2015). LFI FR IQA metrics include the algorithms in Min et al. (2020) and Meng et al. (2020). LFI NR IQA metrics include BELIF (Shi et al., 2019), Tensor-NLFQ (Zhou et al., 2020), and VBLIF (Xiang et al., 2020).

**Table 2:** The performance comparison of classical IQA indexes on three benchmark databases.

	**Database**	**SHU**				**VALID-10bit**				**NBU-LF1.0**				**Overall**	
	**Metric**	**RMSE**	**PLCC**	**SROCC**	**KROCC**	**RMSE**	**PLCC**	**SROCC**	**KROCC**	**RMSE**	**PLCC**	**SROCC**	**KROCC**	**WSROCC**	**MSROCC**
2D-FR	PSNR	0.6316	0.8190	0.8859	0.7315	0.4122	0.9036	0.8868	0.7158	0.5982	0.7627	0.7609	0.5640	0.8383	0.8295
	SSIM	0.6422	0.8121	0.8262	0.6567	0.3481	0.9323	0.9273	0.7614	0.6197	0.7424	0.7223	0.5218	0.8049	0.8253
	MS-SSIM	0.5192	0.8817	0.8909	0.7150	0.3155	0.9447	0.9348	0.7793	0.5447	0.8083	0.8125	0.6078	0.8689	0.8794
	IW-SSIM	0.5129	0.8848	0.8892	0.7181	0.2781	0.9573	0.9441	0.7957	0.5461	0.8071	0.8045	0.6032	0.8668	0.8793
	FSIMc	0.5362	0.8733	0.8928	0.7168	0.2907	0.9533	0.9477	0.8006	0.5351	0.8157	0.8106	0.6055	0.8714	0.8837
	RFSIM	0.5977	0.8397	0.8473	0.6686	0.5738	0.8028	0.7915	0.6006	0.7877	0.5242	0.5352	0.3857	0.7180	0.7247
	NQM	0.6507	0.8065	0.8129	0.6330	0.7043	0.6815	0.6675	0.4867	0.7369	0.6044	0.5938	0.4264	0.7028	0.6914
	GSM	0.6381	0.8148	0.8209	0.6410	0.4159	0.9018	0.8686	0.7139	0.6890	0.6671	0.6583	0.4914	0.7675	0.7826
	VSNR	0.6255	0.8228	0.8408	0.6547	0.5425	0.8260	0.8049	0.6234	0.6199	0.7422	0.7497	0.5497	0.7995	0.7985
	MAD	0.5311	0.8759	0.8652	0.6869	0.2744	0.9585	0.9327	0.7776	0.4798	0.8549	0.8583	0.6614	0.8748	0.8854
	GMSD	0.5353	0.8737	0.8782	0.7003	0.2604	0.9627	0.9465	0.8037	0.5669	0.7902	0.7900	0.5916	0.8569	0.8716
	HDRVDP	0.6668	0.7955	0.7754	0.5935	0.4254	0.8970	0.8799	0.6963	0.7358	0.6059	0.5247	0.3744	0.6987	0.7267
	SFF	0.4594	0.9087	0.9196	0.7597	0.3299	0.9394	0.9245	0.7662	0.5554	0.7997	0.8009	0.6050	0.8752	0.8817
	UQI	0.8322	0.6544	0.6004	0.4424	0.4148	0.9024	0.8578	0.7049	0.7729	0.5493	0.5630	0.4066	0.6329	0.6737
	VSI	0.5755	0.8524	0.8556	0.6819	0.5122	0.8466	0.8191	0.6438	0.7044	0.6481	0.6399	0.4774	0.7666	0.7715
2D-RR	WNISM	0.7477	0.7338	0.7250	0.5578	0.3341	0.9378	0.9394	0.7846	0.8057	0.4911	0.4710	0.3229	0.6670	0.7118
	WBCT	0.7582	0.7248	0.7617	0.5861	0.5122	0.8466	0.8191	0.6438	0.6869	0.6697	0.6393	0.4636	0.7254	0.7400
	Contourlet	0.6985	0.7728	0.7498	0.5812	0.4473	0.8854	0.8704	0.6919	0.6595	0.7012	0.6605	0.4786	0.7376	0.7602
Multi- view FR	MP-PSNR	0.5983	0.8393	0.8599	0.6694	0.3633	0.9260	0.9239	0.7614	0.6885	0.6678	0.6611	0.4799	0.7956	0.8150
	MW-PSNR	0.5970	0.8401	0.8548	0.6658	0.3597	0.9275	0.9219	0.7561	0.6600	0.7007	0.6934	0.5019	0.8054	0.8234
	MW-PSNRreduc	0.6452	0.8101	0.8337	0.6433	0.3833	0.9172	0.9100	0.7369	0.7034	0.6494	0.6492	0.4653	0.7771	0.7976
	3DSwIM	0.5958	0.8408	0.8849	0.7135	0.2762	0.9579	0.9513	0.8185	0.7594	0.5709	0.5506	0.3890	0.7693	0.7956
LFI NR	BELIF	0.4847	0.8985	0.8697	0.6953	0.2431	0.9643	0.9454	0.8211	0.7072	0.6489	0.5983	0.4304	0.7798	0.8045
	Tensor-NLFQ	0.3494	0.9469	0.9392	0.8020	0.3163	0.9476	0.9074	0.7586	0.6603	0.6988	0.6064	0.4318	0.8063	0.8177
	VBLFI	0.4025	0.9354	0.9135	0.7613	0.2268	0.9705	0.9414	0.8042	0.5568	0.7934	0.7439	0.5549	0.8538	0.8663
LFI FR	Min et al., 2020	0.5951	0.8412	0.8460	0.6745	0.3335	0.9380	0.8524	0.7052	0.6843	0.6728	0.6659	0.4773	0.7784	0.7881
	Meng et al., 2020	0.4291	0.9208	0.9067	0.7427	0.2692	0.9601	0.9484	0.8043	0.5823	0.7770	0.7040	0.5133	0.8369	0.8530
	MPFS	0.3436	0.9500	0.9534	0.8183	0.2207	0.9734	0.9599	0.8305	0.4336	0.8833	0.8754	0.6908	0.9248	0.9296

This paper used four IQA indexes to measure the fitting of the degree of objective scores and subjective scores. The Pearson linear correlation coefficient (PLCC) and the RMSE denote the accuracy of correlation between mean opinion scores (MOS) and predict scores. The Spearman rank order correlation coefficient (SROCC) and the Kendall rank order correlation coefficient (KROCC) can measure the prediction monotonicity of IQA metrics.

Table 2 presents the performance of classical objective metrics on SHU, VALID-10bit, and NBU-LF1.0 databases, where the values in bold indicate the best performance. The results show that the proposed MPFS method consistently fits well with MOS in both accuracy and monotonicity over the databases of traditional distortion, compressed distortion, and reconstructed distortion.

It can be seen from Table 2 that the performance of traditional algorithms varies in different databases. Although these three databases contain different distortion types, their effects on angular and spatial information are reciprocal. First of all, some traditional algorithms perform well in the VALID-10bit database. This may be due to the fact that angular and spatial distortions in the VALID-10bit database are evenly distributed. Secondly, although the distortion of the SHU database is not derived from LF processing, it is still difficult to estimate the effects of these distortions on LF contents. For example, traditional algorithms do not take advantages they should have for traditional types of distortion. This is due to the fact that traditional algorithms fail to consider the relationship between the angular and spatial quality. In addition, most objective metrics cannot achieve good results in the NBU-LF1.0 database. This may be due to the complex distribution of angular-spatial distortion, for example, the cross effects of angular-spatial distortion vary greatly in different perspectives.

The performance of the multi-view algorithms is similar to that of the traditional 2D algorithms. They perform well when the distribution of the angular-spatial distortion is not complex, but worse for the NBU-LF1.0 database containing the distortion of reconstructed types. It somewhat indicates that the angular-spatial distortion caused by reconstruction algorithms is more complex.

The NR LF-IQA models were trained with 80% contents from each data set used in this paper, and 20% of contents were used for prediction. The optimal training parameters were obtained by multiple adjustments, and the result of each adjustment was the median value of 1,000 experiments. It can be seen from Table 2 that they achieved preferable results at the first two databases, but perform worse for the reconstruction distortions with complex angular-spatial artifacts.

For the FR LF-IQA, the concept of optimal parallax range of human eyes is introduced into the focus stack to calculate the quality of LF images. Meng et al. (2019) used some camera parameters provided by the EPFL database (Honauer et al., 2016) when calculating the optimal parallax range, while some databases do not have these parameters. Therefore, in combination with the experiments of refocusing factors in section 4.7, we set the focusing range of Meng et al. (2020) as [−3, 3] over all databases for the sake of fairness. Min et al. (2020) computed the quality of LF images through the global–local spatial quality and the angular consistency measurement. It is necessary to note that the angular resolution of all databases is set as 9 × 9 in the comparison experiment for fairness. Therefore, the performance of both Meng et al. (2020) and Min et al. (2020) presented in Tables 2, 3 is not optimal.

**Table 3:** PLCC performance of different distortion types on VALID-10bit, SHU and NBU-LF1.0 databases.

	**Database**	**VALID-10bit**					**SHU**					**NBU-LF1.0**				
**Type**	**Metric**	**HEVC**	**VP9**	**P1**	**P2**	**P3**	**GB**	**JPEG2k**	**JPEG**	**MB**	**WN**	**NN**	**BI**	**EPICNN**	**DR**	**VDSR**
2D-FR	PSNR	0.9522	0.9392	0.9282	0.9361	0.8569	0.9198	**0.9502**	0.9752	0.8674	0.9570	0.7740	0.9345	0.8794	0.7030	0.7176
	SSIM	0.9493	0.9407	0.9531	0.9453	0.9289	0.9133	0.8697	0.9724	0.8446	0.9420	0.7951	0.8654	0.8502	0.4157	0.8415
	MS-SSIM	0.9625	0.9522	0.9444	0.9464	0.9360	0.9070	0.9321	0.9725	0.8983	0.9548	0.7695	0.9083	0.9294	0.6854	0.9056
	IW-SSIM	0.9727	0.9674	0.9567	0.9561	**0.9599**	0.9366	0.9375	0.9688	0.9430	0.9549	0.7409	0.9108	0.9360	0.7219	0.6393
	FSIMc	0.9667	0.9651	0.9569	0.9619	0.9409	0.9394	0.9389	**0.9797**	0.9134	0.9157	0.7810	0.9201	0.9213	0.6561	0.8912
	RFSIM	0.9368	0.9219	0.9220	0.7790	0.8378	0.8057	0.8593	0.9162	0.6631	0.9439	0.9189	0.8742	0.2057	0.8104	0.6917
	NQM	0.7686	0.6794	0.7272	0.6573	0.6725	0.7450	0.8479	0.8890	0.5832	0.9322	0.7128	0.8002	0.6220	0.7248	0.5475
	GSM	0.9761	0.9555	0.9677	0.9367	0.8530	0.8351	0.8257	0.9377	0.5277	0.9316	**0.9507**	0.8943	0.7124	**0.8552**	0.6360
	VSNR	0.8820	0.8273	0.8644	0.8747	0.8119	0.8363	0.6665	0.8758	0.6889	0.8569	0.8079	0.8498	0.8144	0.6629	0.7619
	MAD	0.9793	0.9674	**0.9774**	0.9504	0.9366	0.8769	0.9174	0.9186	0.8498	0.9551	0.9095	**0.9501**	**0.9429**	0.8496	0.8973
	GMSD	0.9782	0.9701	0.9731	**0.9738**	0.9520	0.9210	**0.9637**	0.9716	0.9260	0.9009	0.7216	0.9170	0.9265	0.7432	**0.9314**
	HDRVDP	0.9530	0.8827	0.9135	0.9016	0.8796	0.7197	0.8695	0.9523	0.5510	0.9600	0.8910	0.9418	**0.9396**	**0.8500**	0.7857
	SFF	0.9646	0.9528	0.9646	0.9678	0.8787	0.8799	0.9408	0.9734	0.8470	0.9308	0.7845	**0.9462**	0.9271	0.7273	**0.9466**
	UQI	0.9699	0.9680	**0.9749**	0.8785	0.9207	0.6885	0.4614	0.2193	0.5023	0.8736	0.7082	0.8691	0.1932	0.7449	0.0975
	VSI	0.9669	0.9503	0.9668	0.7954	0.8796	0.8413	0.8489	0.9525	0.5385	0.9378	**0.9355**	0.8994	0.7243	**0.8505**	0.6240
2D-RR	WNISM	0.9651	0.9537	0.9522	0.9282	0.9038	0.8924	0.6937	0.8170	0.8839	0.8508	0.7289	0.6830	0.7778	0.4444	0.8648
	WBCT	0.9128	0.8492	0.9105	0.9079	0.8648	0.8075	0.7910	0.7716	0.7744	0.9101	0.5781	0.8303	0.9144	0.6609	0.8089
	Contourlet	0.9288	0.9007	0.9373	0.9231	0.8498	0.8579	0.8528	0.7922	0.7789	0.9471	0.7039	0.8098	0.9218	0.6773	0.8650
Multi-view FR	MP-PSNR	**0.9818**	0.9766	0.9725	0.9701	0.9508	0.8475	0.8758	0.9391	0.7919	0.8190	0.8414	0.8441	0.6679	0.7210	0.7039
	MW-PSNR	0.9709	0.9619	0.9641	0.9610	0.9435	0.8221	0.8760	0.9622	0.6799	0.9074	0.8137	0.8917	0.6805	0.7601	0.6554
	MW-PSNRreduc	0.9784	0.9760	**0.9749**	0.9626	0.9539	0.7508	0.8706	0.9512	0.6076	0.8345	0.8159	0.8427	0.6096	0.7392	0.6788
	3DSwIM	0.9801	**0.9780**	0.9728	0.9640	0.9459	**0.9522**	0.9458	0.8893	0.9344	0.9139	0.8997	0.8746	0.8392	0.8333	0.8720
LFI NR	BELIF	–	–	–	–	–	0.9045	0.8308	0.9585	0.9388	**0.9665**	0.9026	0.9100	0.7182	0.7520	0.9134
	Tensor-NLFQ	–	–	–	–	–	0.9399	0.9284	**0.9849**	0.9411	**0.9749**	**0.9243**	0.8819	0.8430	0.8096	0.7926
	VBLIF	–	–	–	–	–	**0.9578**	0.7452	0.9694	**0.9632**	**0.9854**	0.8820	0.8905	0.8421	0.7051	0.8885
LFI FR	Min et al., 2020	0.9338	0.9667	0.9540	**0.9801**	**0.9616**	0.9288	**0.9643**	0.9397	0.9534	0.9581	0.7851	0.8303	0.7428	0.7492	0.9219
	Meng et al., 2020	**0.9825**	**0.9739**	0.9665	0.9486	0.9473	**0.9647**	0.8398	0.9772	**0.9815**	0.9586	0.8258	0.8812	0.8758	0.1380	0.9234
	MPFS	**0.9828**	**0.9789**	**0.9765**	**0.9702**	**0.9722**	0.9480	0.9456	**0.9774**	**0.9682**	0.9505	0.8766	**0.9677**	**0.9435**	0.7765	**0.9389**

It should be known that the performance of the same objective algorithm is slightly different in different databases. As suggested in Wang and Li (2010) and Zhang et al. (2014) we analyze the objective IQA metrics with the weighted average results across all databases for the overall performance. The weighted average $\bar{\rho }$ is computed as follows:


(16) $$\begin{array}{l} \bar{\rho }\text{=}\frac{\sum_{i}\rho_{i}\cdot \omega_{i}}{\sum_{i}\omega_{i}} \end{array}$$

where ρ_*i*_ (*i* = 1, 2, 3, 4) is the fitting performance for each database. The weight coefficient of each database depends on the number of distorted images in the respective database. Table 2 presents the overall performance and the ranking of weighted-average SROCC of LF-IQA metrics over all databases.

The last two columns in Table 2 are the weight-average SROCC (WSROCC) and the mean SROCC (MSROCC) for each objective metric over all databases, respectively. It can be seen that MPFS performs much better than the other metrics on the WSROCC and the MSROCC.

### Robustness Against Distortion Types

The robustness of the proposed objective IQA model against various distortion types is verified. Table 3 presents the performance comparison of classical objective models on the abovementioned three databases, covering various distortion types. Specifically, the VALID-10bit database contains two classical video compression schemes and three compression schemes specialized for LF images. The SHU database contains classical compression distortion and display distortion, and the NBU-LF1.0 database contains a variety of reconstructed distortion types specialized for LF images.

In Table 3, the values in bold indicate the first three best PLCC values for each distortion type. The performance of different objective algorithms for different distortion types is analyzed through PLCC, which can reflect the fitting accuracy of two sets of data. The results show that many algorithms have the optimal scope of application, and can only be sensitive to some specific distortion types. For example, most algorithms have a good predicted effect on the compressed distortion types in the VALID-10bit database, but are not effective for the reconstructed distortion types in the NBU-LF1.0 database or the traditional distortion types in the SHU database. The reason may be that the angular and spatial distortion in the VALID-10bit database is evenly distributed, while the cross effects of angular and spatial distortion of the other two databases vary greatly in different perspectives. The proposed method cannot achieve the best prediction for each distortion, but it performs relatively stable for all distortion types. The robustness of MPFS is superior to other metrics.

### The Validity of the MPFS Model

The proposed MPFS method has two applications: the prediction of global angular-spatial distortion and the evaluation of local angular-spatial quality. The prediction framework of global angular-spatial distortion is established on the lenslet images. The angular distortion is first predicted at each macro-pixel. Then, the visual saliency of the central SAI is introduced to combine the angular and spatial information. The evaluation framework of local angular-spatial quality utilized the PC-corner and DoG algorisms to evaluate the loss of the focalizing structure and texture structure on the principal components of the focusing stack, respectively.

Table 4 compares the performance of the proposed MPFS in three cases: only the prediction framework of global angular-spatial distortion, only the local angular-spatial quality framework, and the combination of global and local frameworks. It can be seen that both local and global frameworks are effective in the VALID-10bit database, and they have reverse effects on the other two databases. The local angular-spatial quality evaluation framework based on the focus stack is more effective for both the spatial texture distortion and the focalizing structure loss caused by the angular distortion. Because the global framework is mainly based on the prediction of angular distortion, it will be mediocre when the distribution of the angular-spatial distortion is more complex. But the combination of the two frameworks works well, benefiting from their complementarity. Besides, Table 5 lists the time complexity of the proposed MPFS method. The listed time under each data set is calculated by averaging the run time of all LF images. Although the size of some LF images in the NBU-LF1.0 database is slightly different from those in the other two databases, the running time is similar.

**Table 4:** Performance of individual case on SHU, VALID-10bit, and NBU-LF1.0 databases.

**Database**	**VALID-10bit**		**SHU**		**NBU-LF1.0**	
	**PLCC**	**SROCC**	**PLCC**	**SROCC**	**PLCC**	**SROCC**
Local	0.9662	0.9551	0.8477	0.8286	0.8462	0.8356
Global	0.9493	0.9412	0.8610	0.8633	0.7854	0.7780
Local_Global	0.9734	0.9599	0.9500	0.9534	0.8833	0.8754

**Table 5:** Time complexity on SHU, VALID-10bit, and NBU-LF1.0 databases.

**Database**	**VALID-10bit**	**SHU**	**NBU-LF1.0**
Time (second)	76.4793	74.0516	74.0665

### The Validity of Individual Quality Component

After analyzing the contributions of the local/global angular-spatial quality framework, Table 6 presents various features used in the proposed MPFS algorithm, the first two features measure the loss of focalizing structure and texture structure in the local angular-spatial quality framework. It can be seen that the combination of PC-corner and DoG features can better evaluate the angular-spatial distortion of the focus stack. However, due to the complex distribution of angular-spatial distortion, it does not work well in the SHU database.

**Table 6:** Performance of individual features on SHU, VALID-10bit, and NBU-LF1.0 databases.

**Database**	**VALID-10bit**		**SHU**		**NBU-LF1.0**	
**Features**	**PLCC**	**SROCC**	**PLCC**	**SROCC**	**PLCC**	**SROCC**
PC-corner	0.9643	0.9528	0.8339	0.8203	0.8336	0.8301
PC-corner-DoG	0.9662	0.9551	0.8477	0.8286	0.8462	0.8356
PC-corner-DoG-Y	0.9664	0.9534	0.8896	0.8785	0.8757	0.8684
PC-corner-DoG-YUV	0.9734	0.9599	0.9500	0.9534	0.8833	0.8754

In addition to the PC-corner and DoG features, Table 6 also presents the performance after adding the luminance and chrominance features. These two features improve the accuracy of the evaluation algorithm. It can be seen that the chroma information contributes greatly to improve the performance of the proposed method in the SHU database because of the high chromaticity distortion of JPEG.

### The Impact of Principal Components on the MPFS Model

The order of the principal components of the focus stack is obtained by sorting the eigenvalues and the corresponding eigenvectors of its covariance. The eigenvectors with larger eigenvalues reflect a larger amount of information. As can be seen from Figure 4, the first-order principal component reflects most of the low-frequency information in the focus stack, in which the defocused blur of the focus stack is mainly distributed in the first-order principal component. The other principal components mainly reflect the high-frequency information of the focus stack, and the distortion of focalizing structure is obvious in the higher-order principal components.

**Figure 6:**
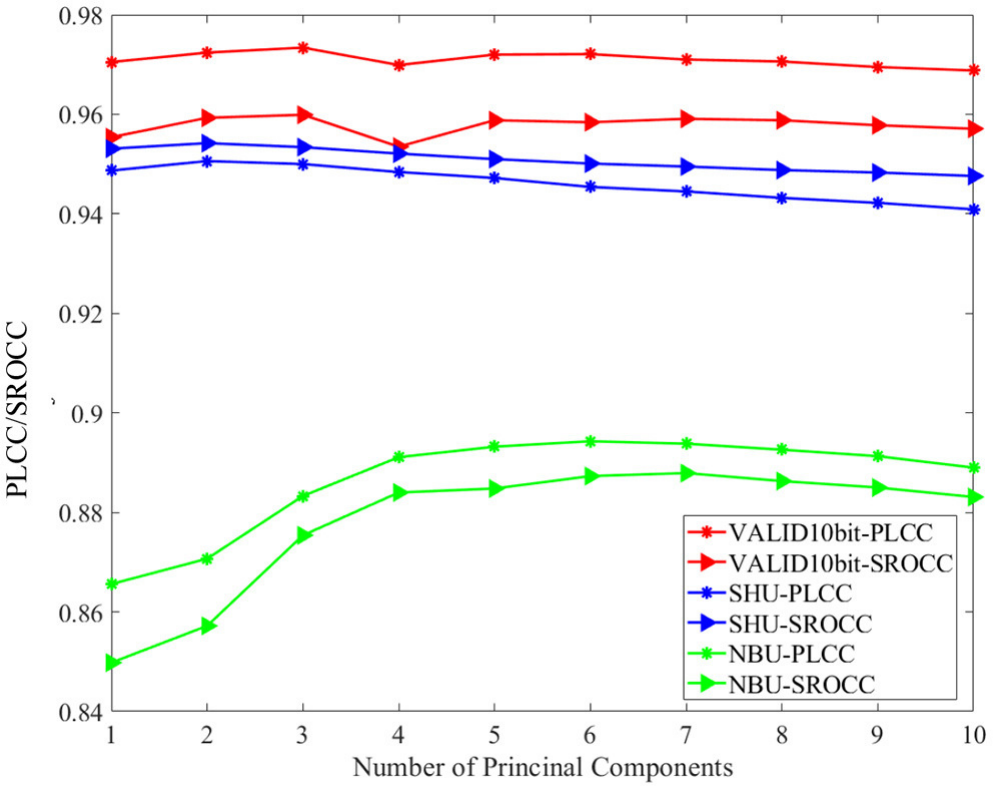
The distribution of Pearson linear correlation coefficient (PLCC)/Spearman rank order correlation coefficient (SROCC) of the MPFS method at different numbers of the principal components in the focus stack over the three databases.

Although the PCA is carried out in the local angular-spatial quality evaluation framework, we analyze the impact of different numbers of principal components on the overall algorithm due to the complementarity of the two frameworks. Figure 6 describes the distribution of PLCC/SROCC of the proposed MPFS method at different numbers of the principal components in the focus stack over the three databases. It can be seen that the variation trend of the final evaluation results over the three databases is inconsistent with an increase of the number of principal components, which is related to the completely different distortion types of the three databases. We finally choose the first three principal components to calculate the local angular-spatial quality for accuracy and simplicity.

### The Impact of Refocusing Factors on the MPFS Model

The evaluation framework of local angular-spatial quality is based on the focus stack, while the refocusing factors will affect the evaluated final results. Specifically, the refocusing factors contain the refocus scope and refocus step. This paper conducts the refocus operation in the spatial domain. The refocused images are obtained by the LFFiltShiftSum function in LFToolbox0.4, which acts on shifting and summing the SAIs within a given slope scope to obtain the focus stack. Different slopes correspond to different depth planes. A step between the two slopes determines the number of refocused images within the given refocus scope.

Table 7 lists the PLCC and SROCC in multiple refocus scopes over the three databases. We set 15 intervals for all to refocus scopes in Table 7, that is, 16 refocus images are obtained. Table 8 illustrates the effect of different intervals on the local angular-spatial quality under the optimal refocused scope in Table 7.

**Table 7:** PLCC and SROCC of different refocus scopes on VALID-10bit, SHU, and NBU-LF1.0 databases.

**Database**	**VALID-10bit**		**SHU**		**NBU-LF1.0**	
**Scope**	**PLCC**	**SROCC**	**PLCC**	**SROCC**	**PLCC**	**SROCC**
[-1, 1]	0.9694	0.9544	0.9475	0.9503	0.8802	0.8727
[-2, 2]	0.9691	0.9560	0.9475	0.9499	0.8802	0.8721
[-3, 3]	0.9734	0.9599	0.9500	0.9534	0.8833	0.8754
[-4, 4]	0.9723	0.9583	0.9473	0.9514	0.8735	0.8582

**Table 8:** PLCC and SROCC of different refocus intervals on VALID-10bit, SHU, and NBU-LF1.0 databases.

**Database**	**VALID-10bit**		**SHU**		**NBU-LF1.0**	
**Scope**	**PLCC**	**SROCC**	**PLCC**	**SROCC**	**PLCC**	**SROCC**
[-3, 3, 10]	0.9655	0.9488	0.9433	0.9475	0.8743	0.8642
[-3, 3, 15]	0.9734	0.9599	0.9500	0.9534	0.8833	0.8754
[-3, 3, 20]	0.9718	0.9583	0.9495	0.9525	0.8765	0.8616

The results show that the optimal refocus scope of the focus stack is [−3, 3] in the local angular-spatial quality evaluation framework, and the optimal number of refocusing intervals is 15. However, the change of the refocus scope and step cannot cause a great influence, which indicates that the local angular-spatial quality framework based on the focus stack is relatively stable.

## Discussion

The quality evaluation for LF images is a new challenge due to the abundant scene information and the complex imaging structure. The existing objective methods are mainly carried out on the classical representations of LF images, especially SAIs, focus stack, and EPIs. It should be noted that different LF representations usually place different emphasis on the distribution of angular and spatial information. Comparatively speaking, the lenslet image and EPIs directly reflect the distortion of angular information, while the focus stack and SAIs directly reflect the distortion of spatial information. The advantages of angular-spatial information distribution in each representation can be better utilized by combining these LF representations, but the disadvantage is increased computational complexity.

The key to quality evaluation of LF images lies on how to combine the human visual perception and the LF angular-spatial characteristics. In this paper, we propose a new LF quality evaluation method through the global angular-spatial quality framework based on macro-pixels and the local angular-spatial quality framework based on the focus stack. The global angular-spatial quality framework evaluates the distortion of luminance and chrominance at each macro-pixel, primarily representing the angular distortion. Then, the visual saliency of human eyes to spatial texture structure is introduced to pool an array of predicted values of angular distortion. However, although the macro-pixel array reflects the global information of LF images, the single macro-pixel lacks the texture information of objects in the scene. Fortunately, the focus stack can help to measure the damage of spatial texture structure and the loss of the focalizing structure caused by the angular distortion. Therefore, a local angular-spatial quality framework based on the principal component of the focus stack is adopted to complement the global framework. The losses of the focalizing structure and texture structure are analyzed through the PC-corner similarity and DoG texture similarity, respectively. Extensive experimental results show that better performance can be obtained by combining the complementary local/global angular-spatial quality evaluation framework.

In the future work, we decide to explore ways to reduce the computational complexity of evaluating global angular-spatial distortion distribution, such as introducing the random sampling mechanism into the distortion prediction of macro-pixels. Moreover, how to achieve better integration of LF angular-spatial characteristics and human visual characteristics under the condition of low computational complexity is still a challenge for the quality evaluation of LF images. The application of human visual characteristics in this paper is divided into two types. First, the global framework uses the saliency distribution of spatial information as the weight to realize the integration of the distribution of angular distortion and spatial structure. Second, feature extraction operators of PC-corner and DoG, which simulate human visual characteristics, are, respectively, applied to the calculation of focalizing structure and texture structure. In general, the application of human visual characteristics in the quality evaluation of LF images mainly lies on the fusion of angular and spatial distortion prediction, or the feature extraction in the prediction of angular distortion and spatial distortion. It is difficult to achieve the perfect fusion of LF angular-spatial characteristics and human visual characteristics in the traditional algorithms, while the deep learning methods have strong ability to learn the relationship between the angular information and spatial information, as well as the relationship between the human visual characteristics and LF angular-spatial characteristics.

## Data Availability Statement

Publicly available datasets were analyzed in this study. This data can be found at: Visual quality Assessment for Light field Images Dataset (VALID), https://mmspg.epfl.ch/VALID.

## Author Contributions

CM performed the experiments and wrote the first draft of the manuscript. PA provided mentorship into all aspects of the research. PA and XH modified the content of the manuscript. All authors contributed ideas to the design and implementation of the proposal, read, and approved the final version of the manuscript.

## Funding

This work was supported in part by the National Natural Science Foundation of China, under (Grant Nos. 62020106011, 62001279, 62071287, and 61901252), Science and Technology Commission of Shanghai Municipality under (Grant 20DZ2290100).

## Conflict of Interest

The authors declare that the research was conducted in the absence of any commercial or financial relationships that could be construed as a potential conflict of interest.

## Publisher's Note

All claims expressed in this article are solely those of the authors and do not necessarily represent those of their affiliated organizations, or those of the publisher, the editors and the reviewers. Any product that may be evaluated in this article, or claim that may be made by its manufacturer, is not guaranteed or endorsed by the publisher.
